# The effectiveness of transnasal high flow nasal cannula in bronchoscopy under sedation: a systematic review and meta-analysis

**DOI:** 10.3389/fmed.2024.1428431

**Published:** 2024-07-10

**Authors:** Chen Wei, Shaoyong Ma, Jingwen Wang, Na Yang, Dandan Wang, Liping Yuan, Yingying Wang

**Affiliations:** ^1^Nursing Department, Yijishan Hospital of Wannan Medical College, Wuhu, Anhui, China; ^2^School of Nursing, Wannan Medical College, Wuhu, Anhui, China

**Keywords:** high flow nasal cannula, conventional oxygen therapy, non-invasive ventilation bronchoscopy, hypoxemia, meta-analysis

## Abstract

**Background:**

The objective of this study was to conduct a systematic review and meta-analysis of the clinical application effects of transnasal high flow nasal cannula compared to other conventional modalities for oxygen therapy devices in patients undergoing bronchoscopy.

**Methods:**

A comprehensive literature search was conducted in multiple English databases, including PubMed, Web of Science, and Cochrane Library, to collect relevant studies on the application of high flow nasal cannula in patients undergoing bronchoscopy, and conducted a meta-analysis utilizing RevMan 5.4 software, following the predetermined inclusion and exclusion criteria.

**Results:**

A total of 12 studies meeting the inclusion criteria were included, involving 1,631 patients (HFNC group: *n* = 811, other oxygen therapy group: *n* = 820). The meta-analysis results demonstrated that HFNC significantly reduced the incidence of hypoxemia and improved the minimum oxygen saturation compared to conventional oxygen therapy (RR = 0.27, 95% CI: 0.18–0.41, *p* < 0.00001; MD = 6.09, 95% CI: 3.73–8.45, *p* < 0.00001). Furthermore, HFNC showed statistically significant differences when compared to non-invasive ventilation in terms of hypoxemia incidence (RR = 3.52, 95% CI: 1.13–10.97, *p* = 0.03) and minimum oxygen saturation (MD = −1.97, 95% CI: −2.97-−0.98, *p* < 0.0001). In addition, HFNC resulted in significantly shorter surgical time and higher PaO2 at the end of the procedure compared to conventional oxygen therapy (MD = 1.53, 95% CI: 0.66–2.40, *p* = 0.0006; MD = 15.52, 95% CI: 10.12–20.92, *p* < 0.00001). However, there were no statistically significant differences observed in PaCO2, EtCO2, and MAP at the end of the procedure (MD = 1.23, 95% CI: −0.74-3.20, *p* = 0.22; MD = −0.35, 95% CI: −3.77-3.06, *p* = 0.84; MD = −0.54, 95% CI: −2.44-1.36, *p* = 0.58).

**Conclusion:**

When HFNC or NIV is utilized during the examination and treatment of bronchoscopy patients, both oxygenation modalities enhance oxygenation function and reduce the incidence of hypoxemia compared to conventional oxygen therapy. HFNC can be regarded as a viable alternative to NIV for specific high-risk patients undergoing bronchoscopy. It decreases the duration of bronchoscopy and improves the PaO_2_ levels at the end of the procedure, but does not significantly impact the PaCO_2_, EtCO_2_, and mean arterial pressure.

**Systematic review registration:**

https://www.crd.york.ac.uk/PROSPERO/, identifier 1414374462@qq.com.

## 1 Introduction

Bronchoscopy is a common invasive procedure performed in the field of respiratory medicine for the diagnosis and treatment of diseases. It involves inserting a flexible tube with a camera into the lower respiratory tract through the mouth or nose, allowing visualization and examination of the trachea and bronchial tubes, including the distal end (1, 2). Sedation is recommended during bronchoscopy to ensure patient comfort, compliance, and reduce anxiety (3). Although painless bronchoscopy is widely practiced, it can still lead to various complications such as coughing, shortness of breath, hypoxemia, and arrhythmia, causing significant discomfort to patients. Hypoxemia, in particular, has an incidence rate ranging from 30 to 70% (4–6).

Conventional oxygen therapy (COT) has limitations in providing sufficient oxygen concentration and meeting the comfort needs of patients, especially those with difficult airways or undergoing prolonged procedures. Hence, selecting an appropriate oxygen therapy regimen is crucial. High flow nasal cannula (HFNC) is a novel noninvasive high-flow oxygen therapy technique that delivers stable temperature, humidity, and a constant inhaled oxygen concentration. It effectively corrects hypoxia and reduces the occurrence of hypoxic respiratory failure in patients (7). While HFNC has been successfully used in critical care settings (8, 9), its efficacy in bronchoscopy remains a matter of debate and lacks comprehensive systematic evaluation. In recent years, studies such as Irfan et al. (6) have demonstrated a significant decrease in the incidence of hypoxemia and an improvement in minimum SpO_2_ during bronchoscopy when HFNC was used compared to COT. However, another study reported no cases of hypoxemia in either the HFNC or COT groups (10). Additionally, when comparing HFNC to non-invasive ventilation (NIV), the incidence of hypoxemia was significantly lower in both groups (11), suggesting that HFNC may offer advantages over NIV in terms of reducing hypoxemia.

The operative time was notably reduced in the HFNC group in two studies (4, 11), and HFNC contributed to an improvement in the rise of partial pressure of oxygen (PaO_2_) in studies (10, 12). However, there was no observed improvement in partial pressure of carbon dioxide(PaCO_2_), end-tidal CO_2_ (EtCO_2_) and mean arterial pressure (MAP). Currently, HFNC is rapidly gaining prominence and offers numerous advantages. However, the majority of studies, both nationally and internationally, investigating the application of HFNC during bronchoscopy have been conducted in single-center settings with limited sample sizes. Furthermore, there is a lack of consistency in the outcome measures reported across the studies. Published meta-analyses (13, 14) have investigated the benefits of HFNC during bronchoscopy; however, there is a dearth of relevant comparisons with NIV, and the literature is rapidly evolving in this area. As a result, this study utilized meta-analysis to assess the impact of HFNC in bronchoscopy, with the aim of providing a reference for the adoption of HFNC in clinical bronchoscopy patients.

## 2 Information and methods

### 2.1 Data source and search

A computerized search of PubMed, Web of Science, and Cochrane Library literature databases was conducted with a timeframe of construction to April 2024 for relevant literature on the application of HFNC in patients undergoing bronchoscopy. The following Medical Subject Headings (MeSH) were used in the search: (‘high flow nasal cannula’ OR ‘HFNC’ OR ‘high flow oxygen therapy ‘OR ‘HFNO’ OR ‘high flow nasal oxygenation’) AND (‘Bronchoscopy ‘OR ‘Bronchoscope’ OR ‘flexible bronchoscopy’ OR ‘Bronchoscopic ‘OR ‘Bronchoscopic Surgeries’). No language restrictions were imposed. This systematic review was registered in the online PROSPERO International Prospective Register of Systematic Reviews of the National Institute for Health Research (registration number CRD42024540403).

### 2.2 Inclusion criteria

The study object includes patients undergoing bronchoscopy: ① Study object: patients undergoing bronchoscopy; ② Intervention method: comparison of the application effect between HFNC and other oxygen therapy modalities in patients undergoing bronchoscopy; ③ Type of study: randomized controlled trial (RCT); ④ Outcome indexes: incidence rate of hypoxemia, minimum SpO_2_, procedure time, PaCO_2_ at the end of the examination, PaO_2_, EtCO_2_, and MAP.

### 2.3 Exclusion criteria

The exclusion criteria included the following: ① Literature in the form of reviews, conference abstracts, case reports, and other non-original research articles; ② Incomplete or missing extracted data; ③ Unavailability of full-text articles.

### 2.4 Literature screening and extraction

In accordance with the inclusion and exclusion criteria, two reviewers trained in evidence-based nursing independently reviewed and evaluated the relevant literature, collected and categorized the study data, and conducted comparative verification. In the event of discrepancies, discussions were held, and if necessary, a third party was consulted for adjudication. Studies that did not meet the inclusion criteria were excluded, and data including article author, title, publication time, intervention, number of cases, various outcome indicators, and risk of bias were extracted and summarized in an Excel sheet.

### 2.5 Quality evaluation

This study underwent a quality assessment by two researchers utilizing evidence-based care principles in accordance with the Cochrane Handbook of Systematic Evaluation criteria for appraising randomized controlled trials. The assessment covered seven key areas: generation of randomized sequences, allocation concealment, blinding of study subjects or interveners, blinding of outcome measures, data completeness, selective reporting of results, and other biases. Each outcome was categorized as “low risk,” “high risk,” or “unclear.” The quality of the original literature was graded as A if it fully met the specified criteria, B if it partially met the criteria, and C if it did not meet the criteria at all.

### 2.6 Statistical processing

The meta-analysis was conducted using Review Manager 5.4 software, with mean difference (MD) and 95% confidence interval chosen as the effect indicators. The selection of the model depended on the homogeneity of the data: the fixed effect model was used when the data were homogeneous (*p* ≥ 0.1 and *I^2^* < 50), while the random effect model was chosen for heterogeneous data (*p* < 0.1 and *I^2^* ≥ 50%). The combined results of the effect indicators were assessed using the Meta-analysis test criterion, indicating statistical significance when *p* < 0.05.

## 3 Results

### 3.1 Literature search results and basic characteristics of included literature

**Figure 1:**
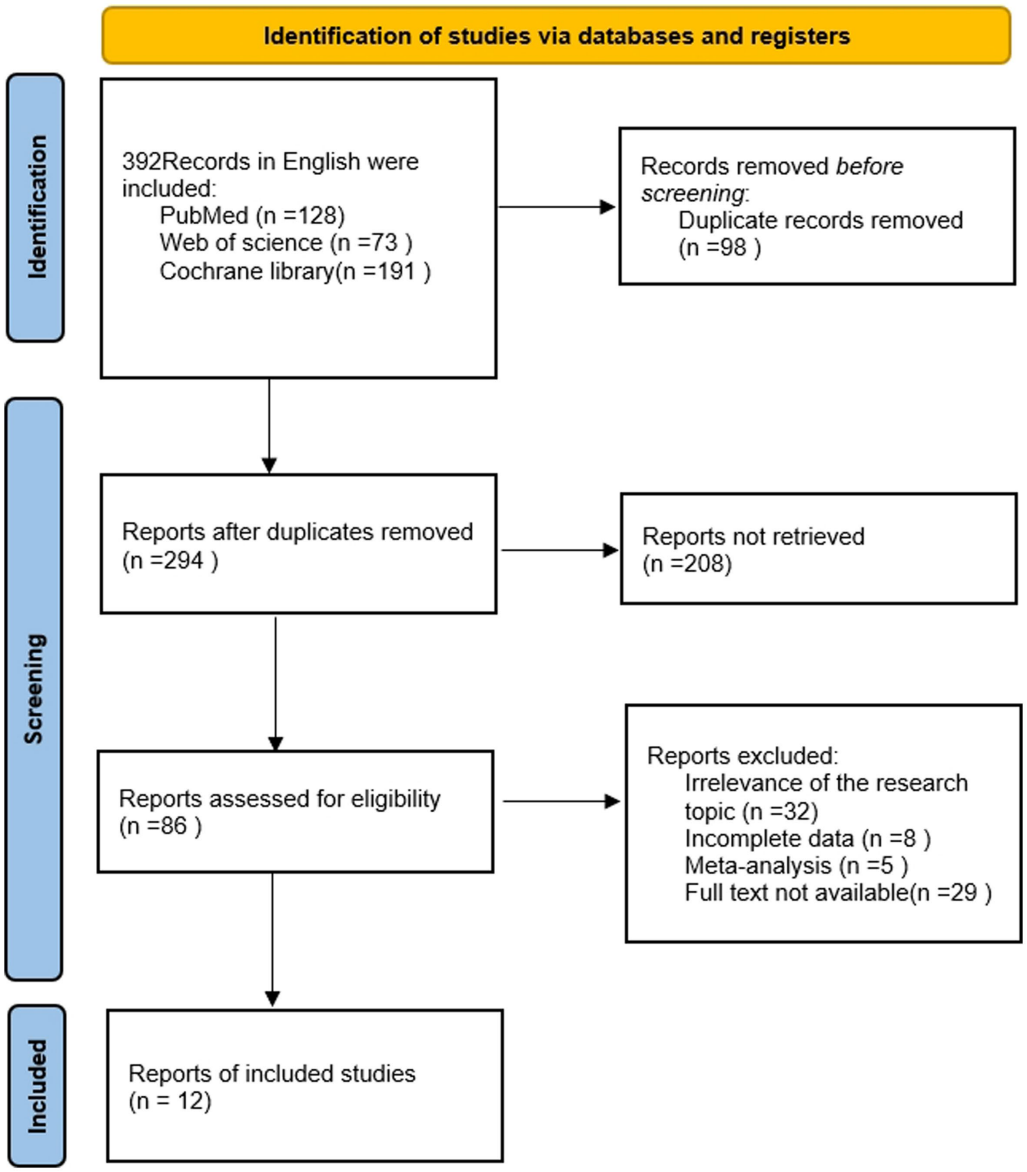
Basic feature table of articles.

In this study, the literature search was conducted in accordance with the specified criteria, resulting in a total of 392 English-language publications being identified, including 128 from PubMed, 73 from Web of Science, and 191 from Cochrane. Following the review of titles and abstracts, 12 RCTs (4–6, 10–12, 15–20) were included in the study. The RCTs comprised 811 cases in the HFNC group and 820 cases in the COT group. The difference between the two groups was not statistically significant (*p* > 0.05) and was considered comparable. The selected studies mainly focused on bronchoscopy, flexible bronchoscopy, bronchial ultrasonography, and fiberoptic bronchoscopy. In all studies, bronchoscopy was performed by using a local anesthetic (lidocaine) sprayed over the entire scopal passage (e.g., oropharynx, airway), together with different combinations of the following sedation: short-acting opioids (fentanyl/alfentanyl/remifentanil) (4, 6, 10, 12, 15, 20), intravenous propofol (4, 10, 12, 18) and midazolam (5, 6, 10, 12, 15, 17). The depth of sedation was titrated to pre-defined clinical endpoints (Ramsay sedation scale, modified observer’s alertness/sedation scale, sedation state scale, Richmond Agitation Score); EEG monitoring (bispectral index); or society-based recommendations (e.g., ASA guidelines) in all the included trials. A visual representation of the search process can be found in Figure 1, and the basic characteristics of the included literature are detailed in Table 1.

**Table 1:** Basic characteristics of articles.

		Quality assessment					
First author	Country/Chronicity	Samplesize (T/C)	Mean age (year)		Intervention		Outcome
			HFNC	Other Oxygen Therapy	HFNC	Other Oxygen Therapy	
Ben-Menachem (4)	Australia	37/39	54.9 ± 11.7	55.8 ± 11.9	30 L/min	4 L/min	①②③
Douglas (5)	Australia	30/30	62.8 ± 14.1	63.4 ± 14.3	30-70 L/min	10 L/min	①②③⑥
Irfan (6)	United Kingdom	20/20	61.9 ± 12.0	64.5 ± 14.0	HFNC	nasal cannula	①②④⑥
Longhini (12)	Italy	18/18	-	-	60 L/min	2 L/min	①②④⑤
Lucangelo (10)	Italy	15/15	69.45 ± 13.09	65.82 ± 5.73	60 L/min	40 L/min	③④⑤⑦
Saksitthichok (15)	Thailand	26/25	60.0 ± 15.3	57.2 ± 16.7	40 L/min	NIV	①②
Simon (18)	Germany	20/20	64.0 ± 12.0	68.0 ± 11.0	50 L/min	NIV	①②
Yilmazel Ucar (19)	Turkey	85/85	57.8 ± 14.0	57.5 ± 14.0	35 L/min	nasal cannula	①⑦
Wang (11)	China	392/396	58.11 ± 11.91	57.60 ± 13.39	50 L/min	6 L/min	①②⑦
Sharluyan (16)	Spain	51/63	4.53 ± 5.01	4.20 ± 5.28	-	-	③
Zhang (20)	China	87/89	64.2 ± 9.3	63.6 ± 7.7	60 L/min	6 L/min	①
Sharma (15)	India	30/30	62.5 ± 7.4	62.7 ± 6.4	40 L/min	3-5 L/min	①②③

HFNC, High flow nasal cannula; NIV, non-invasive ventilation; T, Test group; C, Control group; Endpoint indicators: ① incidence of hypoxemia; ② minimum SpO_2_; ③ Surgical time; ④ PaCO_2_ at the end of the surgery; ⑤ PaO_2_ at the end of the surgery; ⑥ EtCO_2_ at the end of the surgery; ⑦ MAP at the end of the surgery.

### 3.2 Quality evaluation

**Figure 2:**
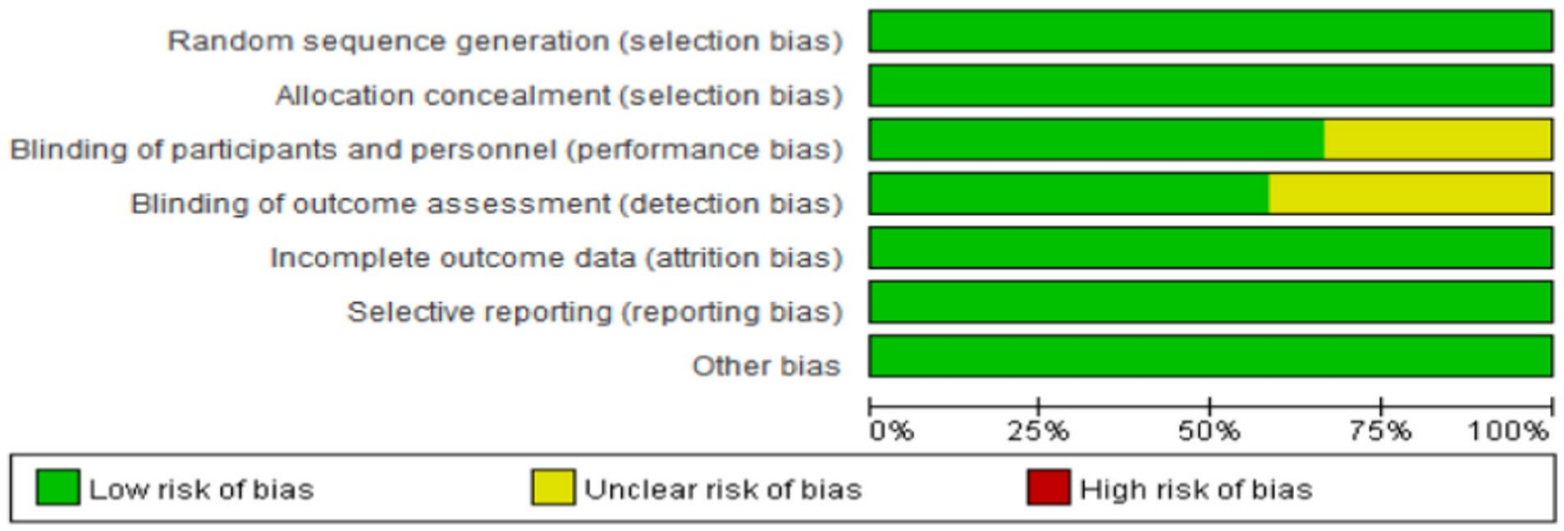
Graph for risk of bias.

A total of 12 studies were included in this study, and the risk of bias was evaluated following the guidelines outlined in the Cochrane Handbook. Among the included studies, 7 were rated as grade A in terms of quality, while the remaining 5 were rated as grade B. The overall risk of bias assessment for the included studies is depicted in Figure 2.

### 3.3 Meta-analysis results

#### 3.3.1 Incidence of hypoxemia

**Figure 3:**
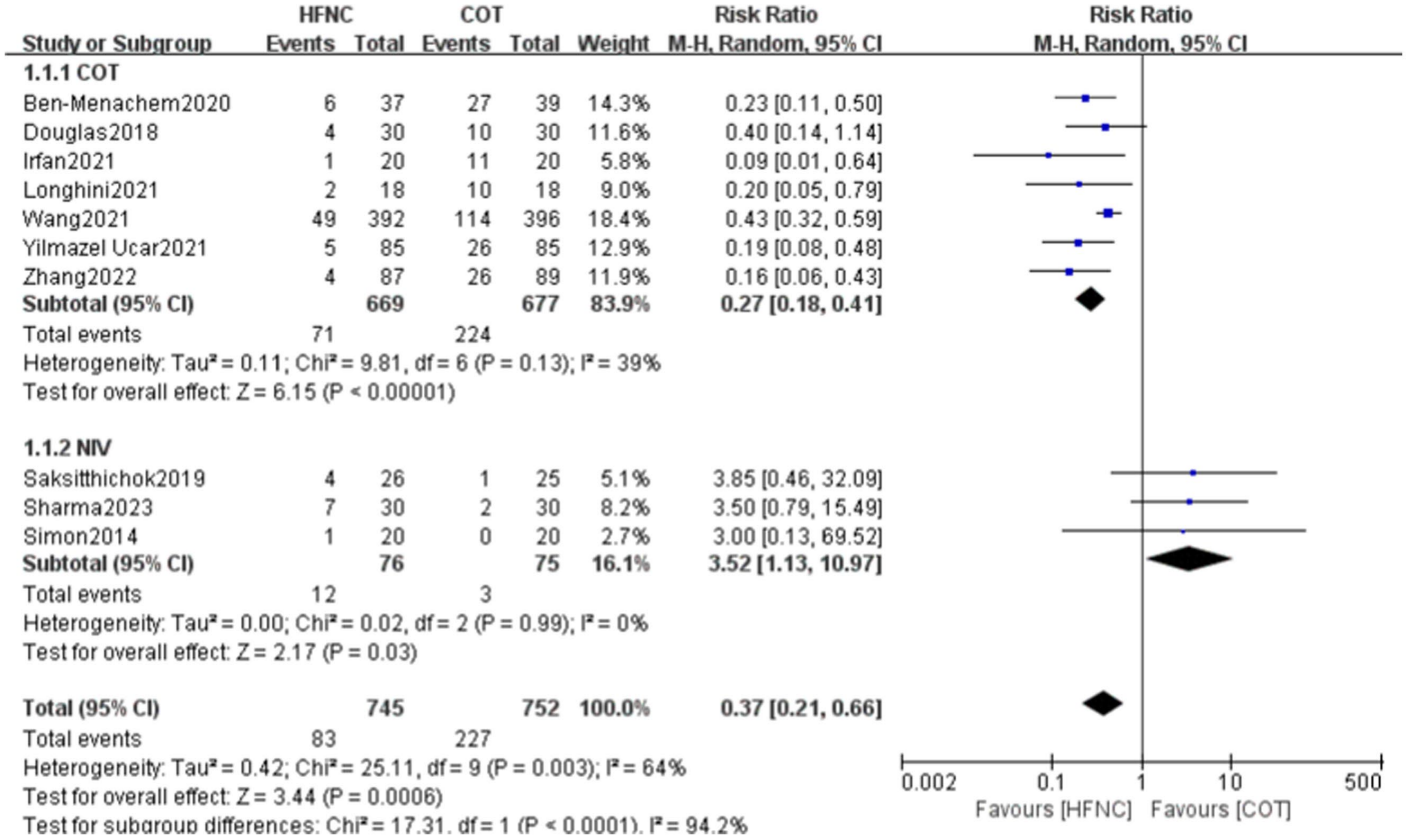
Forest plot comparing the Incidence of hypoxemia in HFNC versus control groups.

A total of 10 papers, involving 1,497 patients (HFNC group: *n* = 745, COT group: *n* = 752), reported the incidence of hypoxemia. Heterogeneity was observed between the two groups, and a random-effects model was used for analysis. The incidence of hypoxemia was significantly lower in the HFNC group compared to the COT group. However, the incidence of hypoxemia was relatively higher in the HFNC group compared to the NIV group. Subgroup analyses were performed by dividing the control group into COT and NIV based on the oxygen therapy used during bronchoscopy. The subgroup analysis showed a statistically significant difference between the two groups for COT (RR = 0.27, 95% CI: 0.18–0.41, *p* < 0.00001) and NIV (RR = 3.52, 95% CI: 1.13–10.97, *p* = 0.03; Figure 3).

#### 3.3.2 Minimum SpO_2_

**Figure 4:**
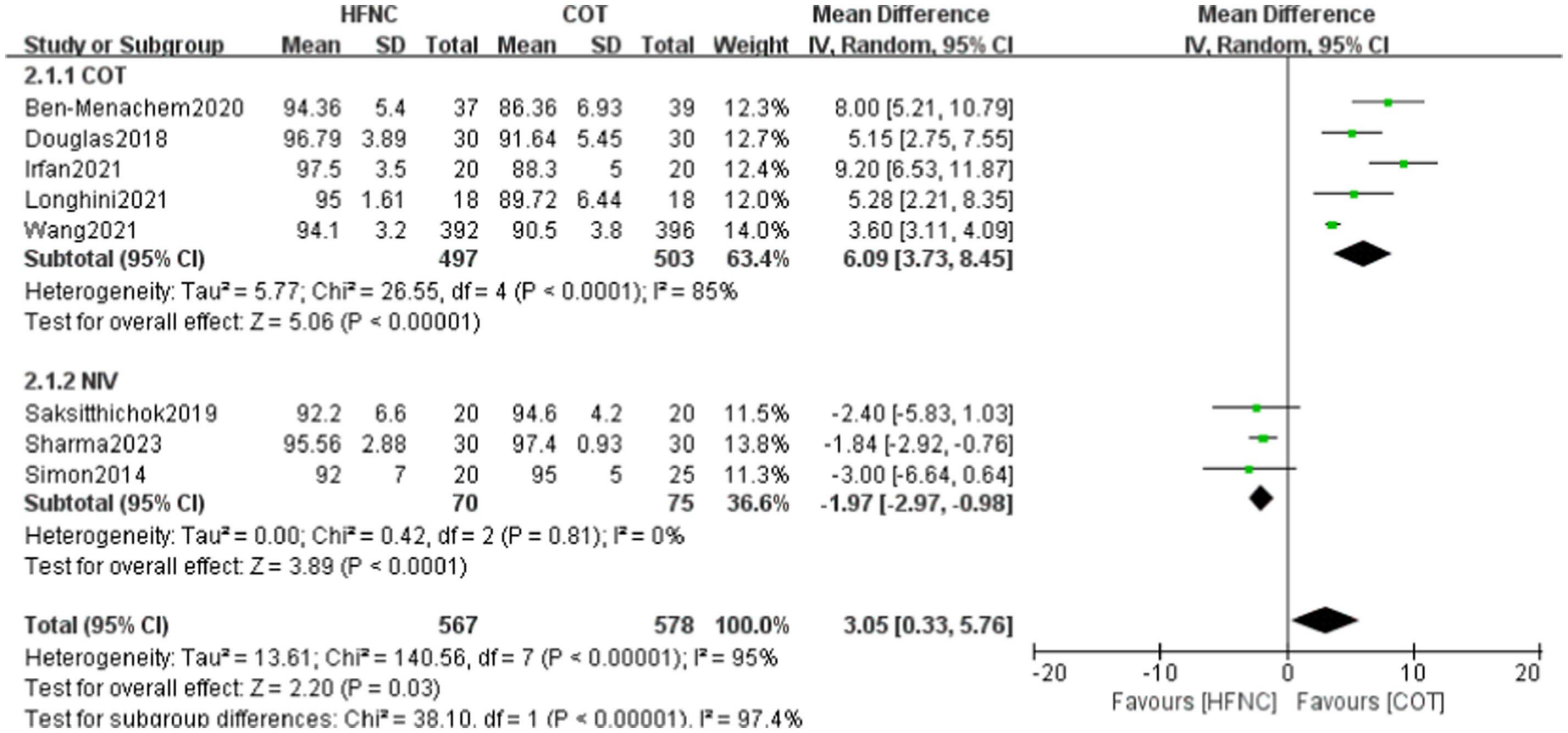
Forest plot comparing Minimum SpO_2_ in HFNC versus control groups.

Eight studies reported the lowest SpO_2_ levels in a total of 357 patients, with 175 individuals in the HFNC group and 182 in the COT group. Upon assessing the study data for heterogeneity, significant variability was observed between the two groups, prompting the selection of a random-effects model. The HFNC group exhibited significantly higher values compared to other oxygen therapies (MD = 3.05, 95% CI: 0.33–5.76, *p* = 0.03). Subgroup analyses were conducted based on the division of oxygen therapy in the control group during bronchoscopy into COT and NIV categories. The study revealed that the lowest SpO_2_ was notably higher in the HFNC group compared to the COT group and lower than the NIV group. In subgroup analysis, statistically significant differences were observed between the HFNC and COT groups (MD = 6.09, 95% CI: 3.73 to 8.45, *p* < 0.00001) as well as between the HFNC and NIV groups (MD = -1.97, 95% CI: −2.97 to 0.98, *p* < 0.0001; Figure 4).

#### 3.3.3 Surgical time

**Figure 5:**
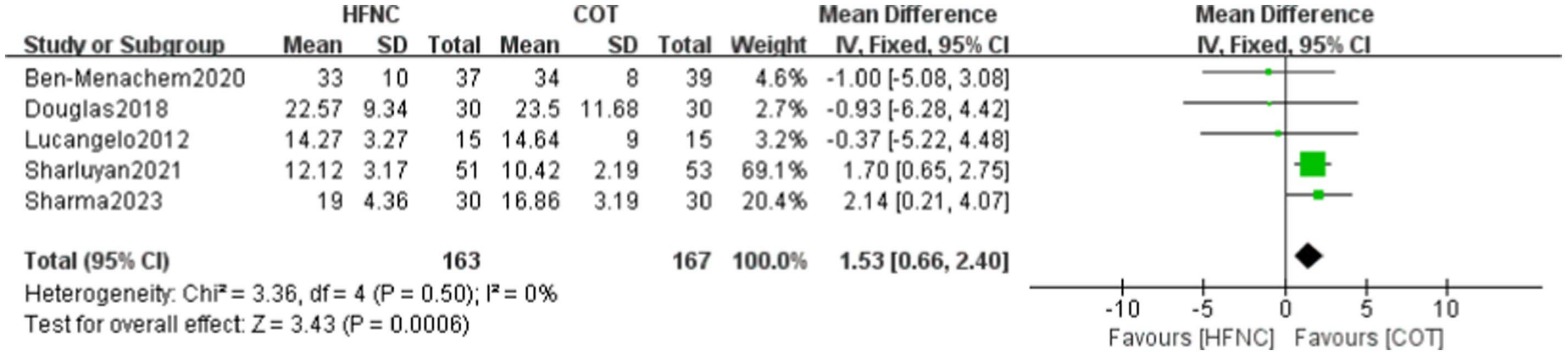
Forest plot comparing Surgical time in HFNC versus control groups.

A total of five papers reported on the operation time, involving 330 patients, with 163 in the HFNC group and 167 in the COT group. The data were assessed for heterogeneity (*p* = 0.50, *I*^2^ = 0%) and showed homogeneity between the two groups. Thus, a fixed effect model was employed for the analysis. The results revealed a statistically significant reduction in the operation time within the HFNC group compared to the COT group (MD = 1.53, 95% CI: 0.66–2.40, *p* = 0.0006; Figure 5).

#### 3.3.4 PaCO_2_ at the end of surgery

**Figure 6:**
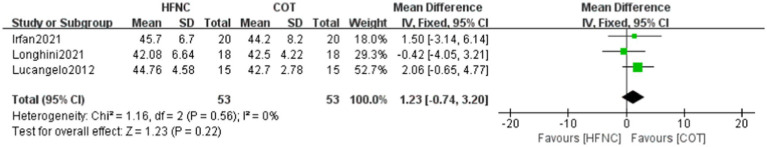
Forest plot comparing PaCO_2_ at the end of surgery in HFNC versus control groups.

PaCO_2_ levels at the conclusion of surgery were documented in three papers, encompassing a total of 106 patients, with 53 individuals in the HFNC group and 53 in the COT group. Analysis of the study data for heterogeneity (*p* = 0.56, *I^2^* = 0%) revealed homogeneity between the two groups, leading to the selection of the fixed effect model for analysis. Meta-analysis results showed that (MD = 1.23, 95% CI: −0.74-0.22, *p* = 0.22), signifying that the observed difference was not statistically significant (Figure 6).

#### 3.3.5 PaO_2_ at the end of surgery

**Figure 7:**
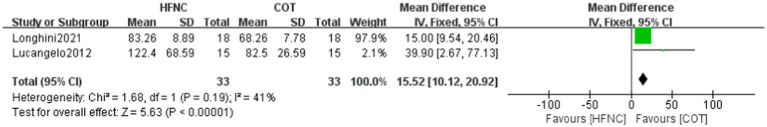
Forest plot comparing PaO_2_ at the end of surgery in HFNC versus control groups.

Two studies reported the lowest PaO_2_ levels, involving a total of 235 patients, with 116 in the HFNC group and 119 in the COT group. Analysis of the study data for heterogeneity (*p* < 0.19, *I*^2^ = 41%) indicated homogeneity between the two groups, leading to the selection of the fixed-effect model for analysis. The lowest SpO_2_ in the HFNC group was lower than that in the COT group(MD = 15.52, 95% CI: 10.12–20.92, *p* < 0.00001), demonstrating a statistically significant difference (Figure 7).

#### 3.3.6 EtCO_2_ at the end of surgery

**Figure 8:**
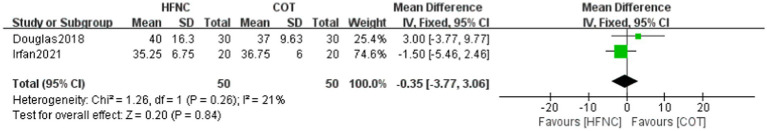
Forest plot comparing EtCO_2_ at the end of surgery in HFNC versus control groups.

EtCO_2_ levels at the conclusion of surgery were reported in two papers, encompassing a total of 100 patients, with 50 in the HFNC group and 50 in the COT group. Analysis of the study data for heterogeneity (*p* = 0.26, *I*^2^ = 21%) revealed homogeneity between the two groups, leading to the selection of the fixed-effect model for analysis. Meta-analysis results showed that (MD = −0.35, 95% CI: −3.77-3.06, *p* = 0.84), signifying that the observed difference was not statistically significant (Figure 8).

#### 3.3.7 Map at the end of surgery

**Figure 9:**
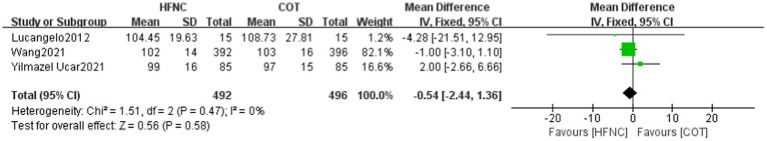
Forest plot comparing MAP at the end of surgery in HFNC versus control groups.

MAP levels at the conclusion of surgery were reported in three papers, encompassing a total of 988 patients, with 492 in the HFNC group and 496 in the COT group. Analysis of the study data for heterogeneity (*p* = 0.47, *I*^2^ = 0%) revealed homogeneity between the two groups, leading to the selection of the fixed effect model for analysis. Meta-analysis results showed that (MD = −0.54, 95% CI: −2.44-1.36, *p* = 0.58), signifying that the observed difference was not statistically significant (Figure 9).

## 4 Discussion

Bronchoscopy is widely recognized as a crucial diagnostic and therapeutic tool for respiratory diseases, with its usage expanding rapidly in clinical settings (13). The literature reviewed in this study utilized lidocaine for local anesthesia, which offers the advantage of being a straightforward and easily administered agent. It exhibits a rapid onset of action and minimally affects the patient’s spontaneous breathing. Additionally, certain studies employed various sedative drugs, primarily fentanyl, propofol, and midazolam, among others. In both groups, multiple sedative drugs were administered at comparable doses and using similar methods, resulting in no significant difference in the level of sedation achieved. This standardized approach to local anesthesia and sedation ensures consistency across the studies and strengthens the validity of the findings in the context of bronchoscopy procedures. The findings of this study demonstrated that HFNC usage resulted in a significant reduction in the incidence of hypoxemia among patients undergoing bronchoscopy, while maintaining relatively high levels of minimum SpO_2_ during the examination and PaO_2_ at the end of the procedure. Additionally, the procedure time was significantly shortened. However, it is important to note that the HFNC group exhibited a higher incidence of hypoxemia and relatively lower minimum SpO_2_ when compared to the NIV group. Nevertheless, there were no significant differences observed in PaCO_2_, EtCO_2_, and MAP levels at the conclusion of the bronchoscopy procedure. HFNC has been suggested as a more effective method of oxygen delivery compared to COT during endoscopic procedures (21). Despite the utilization of HFNC in bronchoscopy, the number of studies conducted remains limited, with a small sample size and low-quality literature. Consequently, the current evidence is insufficient, and further investigation is necessary to evaluate the therapeutic effect of HFNC and address other related issues. Moving forward, additional high-quality, multicenter studies should be conducted to comprehensively assess the effectiveness of HFNC in bronchoscopy.

### 4.1 Effect of HFNC on the incidence of hypoxemia in patients

During bronchoscopy, the insertion of a catheter into the trachea can cause airway narrowing and inadequate ventilation, resulting in subsequent hypoxia and hypoxemia (22, 23), with an incidence ranging from 30 to 70% (4–6). This study included 10 randomized controlled trials (RCTs), all of which reported statistically significant findings when comparing the outcomes with both COT and NIV. Subgroup analysis was conducted based on the mode of oxygen therapy employed in the control group. The analysis revealed a significant reduction in the incidence of hypoxemia during bronchoscopy in the HFNC group compared to the COT group. However, it is important to note that the HFNC group exhibited a higher incidence of bronchoscopic hypoxemia when compared to the NIV group. Notably, Irfan et al. (6) demonstrated a significantly lower incidence of hypoxemia during bronchoscopy when HFNC was utilized in comparison to COT. The findings of the current study align with these results. It should be noted that one study employed an oxygen flow rate of 40 L/min in the COT group and 60 L/min in the HFNC group during bronchoscopy. This comparison is noteworthy because both groups utilized higher oxygen flow rates while maintaining FiO_2_ at 50%, resulting in further improvement in patient oxygenation (10). Notably, HFNC has been shown to enhance pulmonary conductivity and compliance, inhibit bronchoconstriction, and reduce metabolic oxygen consumption. Consequently, both the HFNC and COT groups did not experience hypoxemia. HFNC achieves this by delivering high-flow-rate oxygenation, effectively flushing out residual CO_2_ in the airway, and increasing the oxygen concentration in the patient’s inhaled gas (24). However, it is important to highlight that the incidence of hypoxemia was higher in the HFNC group when compared to NIV, despite demonstrating improvement when compared to the overall incidence in the COT group. This finding can be attributed to the fact that NIV enhances pulmonary ventilation function and alleviates dyspnea symptoms, thereby effectively addressing hypoxia and hypoxemia. Sharma et al. (17) conducted a study in which the NIV group employed a fraction of inspired oxygen (FiO_2_) level of 30%. Furthermore, the ventilation parameters were set to a positive end-expiratory airway pressure of 4 cm H_2_O and a pressure support of 8 cm H_2_O. These settings were adjusted to achieve a tidal volume of 8 mL/kg of ideal body weight and to maintain a minimum peripheral capillary oxygen saturation (SpO_2_) of ≥94%. In the HFNC group, an oxygen flow rate of 40 L/min was employed to maintain a minimum peripheral capillary SpO_2_ of ≥94%. Notably, the results indicated a significant reduction in the incidence of hypoxemia when NIV was utilized, aligning with the findings of the current study. Additionally, a study by Simon (18) demonstrated that the NIV group exhibited significantly improved PaO_2_/FiO_2_ ratios after 15 min of treatment and throughout the bronchoscopy procedure compared to other modalities. NIV was superior to HFNC in maintaining adequate oxygenation before, during and after bronchoscopy in patients with acute hypoxaemic respiratory failure. This superiority can be attributed to the continuous positive airflow provided by NIV, which aids in the re-expansion of collapsed alveoli, leading to improved oxygenation and ventilation. Simultaneously, it alleviates the workload on respiratory muscles and reduces the patient’s respiratory power consumption (8). Moreover, a previous meta-analysis reported that NIV attained a greater nadir value of SpO_2_ during bronchoscopy but similar PaO_2_, desaturation episodes, and PaCO_2_, as compared with HFNC (25).

### 4.2 Effect of HFNC on patients’ minimum SpO_2_

In this study, eight randomized controlled trials were included, all of which demonstrated a statistically significant result indicating that the lowest peripheral capillary oxygen saturation (SpO_2_) during bronchoscopy was higher in the HFNC group compared to the COT group, regardless of the specific procedure conducted. Ben-Menachem et al. (4) conducted a study focusing on the lowest SpO_2_ during bronchoscopy in post-lung transplantation patients. The findings revealed a significant elevation in the lowest SpO_2_ during bronchoscopy, suggesting that HFNC holds promising potential for improving outcomes in lung transplant patients undergoing invasive bronchoscopy. HFNC is a non-invasive oxygen delivery method that mimics the natural respiratory flow rate, resulting in continuous and stable oxygen delivery. This approach has minimal impact on patients’ normal respiratory physiology and offers several benefits, including improved oxygenation and ventilation, enhanced respiratory function, and increased respiratory capacity (24, 26). Moreover, HFNC provides airway humidification, facilitating sputum discharge and alleviating airway obstruction symptoms. Additionally, the warm humidification of the airway enhances lung tissue elasticity, reduces respiratory muscle oxygen consumption and metabolic demands, and improves oxygen supply. Consequently, HFNC effectively addresses dyspnea symptoms (27). Notably, the lowest peripheral capillary oxygen saturation (SpO_2_) levels in the NIV group were higher than those observed in the HFNC group. In patients with respiratory failure undergoing bronchoscopy, NIV demonstrated further improvement in the PaO_2_/FiO_2_ ratio when compared to the COT group (28). Previous studies have consistently reported significant enhancements in intraoperative SpO_2_ and PaO_2_/FiO_2_ ratios with the use of NIV during fiber optic bronchoscopy. This technique proves effective in preventing and ameliorating bronchoalveolar lavage-associated hypoxemia, offering a safe and reliable approach. Notably, the findings of the present study align with previous research (29, 30). In a study by Simon (18), patients undergoing bronchoscopy were divided into two groups based on the mode of oxygen delivery: HFNC and NIV. The results indicated that both oxygenation indices in the NIV group were significantly superior to those in the HFNC group. However, the NIV group exhibited poor patient tolerance, whereas none of the participants in the HFNC group discontinued treatment due to intolerance. NIV provides positive airway pressure ventilation, preventing alveolar atrophy and reducing the work of breathing. However, it is crucial to ensure proper mask fit to the patient’s face, as some individuals may experience intolerance. Additionally, mask occlusion may hinder the physician’s operation and increase friction between the fiberscope and the mask, potentially leading to fiberscope damage. Furthermore, NIV usage may trigger various adverse effects, including dry mouth, facial pressure injury, abdominal distension, air leakage, pneumothorax, and severe hypoxemia (11, 31).

### 4.3 Effect of HFNC on patients’ surgical time

In the study conducted by Wen Zhang et al. (20), the bronchial surgical time in both groups was approximately 5 min, which is relatively short. However, this duration difference of less than 30 s may be influenced by various factors such as patient-related variables and operator expertise. Consequently, after excluding this particular study, the analysis revealed a statistically significant reduction in procedure duration in the HFNC group compared to the COT group. The duration of the included studies varied, ranging from 10 min to 34 min due to variations in surgical procedures. Notably, the study by Li and Samar et al. (32, 33) reported a significantly shorter procedure time in the HFNC group, corroborating the findings of this paper. However, when considering overweight and obese patients undergoing painless bronchoscopy, who were divided into six groups based on different oxygen flow rates, it is important to note that higher oxygen flow rates generally resulted in relatively shorter procedure times. Nevertheless, this correlation was not strictly positive, and the results from this study were not statistically significant (34). These inconsistent findings may be influenced by various factors, including sample size, study design, type of procedure, and intervention.

### 4.4 Effect of HFNC on patients’ PaO_2_ at the end of surgery

This meta-analysis revealed a statistically significant improvement in PaO_2_ at the end of surgery when using HFNC compared to COT. Longhini and Lucangelo et al. (10, 12) conducted a study in which HFNC demonstrated a significant improvement in patients’ oxygenation status at the conclusion of bronchoscopy when compared to COT. During bronchoscopy, the COT group experienced a decrease in PaO_2_ by 10–20%, whereas the HFNC group did not exhibit a significant decline in oxygenation. This observation suggests that HFNC effectively enhances pulmonary ventilation, protects the airway, and reduces the metabolic rate. Consequently, it corrects hypoxic symptoms and improves blood gas indices (35, 36). These findings suggest that HFNC plays a beneficial role in maintaining optimal oxygenation while safeguarding the patient’s ventilation and airway. This, in turn, improves respiratory function and overall oxygenation status while reducing procedure duration. These observations further reinforce the advantages of utilizing HFNC in bronchoscopy. Nevertheless, additional studies are necessary to further investigate the safety and efficacy of HFNC in this context.

### 4.5 Effect of HFNC on patients’ PaCO_2_ and EtCO_2_ at the end of the surgery

This meta-analysis revealed that there was no statistically significant difference in PaCO_2_ and EtCO_2_ levels at the conclusion of bronchoscopy when comparing HFNC with COT (*p* > 0.05). These findings align with previous studies comparing the two oxygen therapy modalities in painless fiberoptic bronchoscopy (37). Additionally, no difference in PaCO_2_ levels at the end of bronchoscopy was observed between the two patient groups. Conversely, a study demonstrated that the utilization of HFNC during bronchoalveolar lavage facilitated sputum expulsion, foreign body removal, and alleviation of respiratory obstruction symptoms (38). Furthermore, the application of HFNC resulted in a further reduction in metabolic consumption of lung tissues, improved oxygen supply and blood gasses, leading to the alleviation of respiratory symptoms and enhanced therapeutic efficacy. In fact, all post-treatment PaCO_2_ levels were lower than those observed in the control group (39). Previous studies have consistently shown the effectiveness of HFNC in minimizing CO_2_ retention (40). Furthermore, the impact of oxygen therapy modality on EtCO_2_ was not significant in the two groups in the studies conducted by Douglas and Irfan (5, 6). In our meta-analysis, several clinical factors introduced heterogeneity in the trials, suggesting potential differences in the response of various groups to different oxygen therapy modalities. The duration and oxygen flow rate of the modality were identified as influential factors. As a result, further evidence is warranted to evaluate the effectiveness of CO_2_ elimination with HFNC.

### 4.6 Effect of HFNC on patients’ MAP at the end of surgery

This meta-analysis revealed no statistically significant difference in MAP at the end of bronchoscopy between HFNC and COT. In a study conducted by Lucangelo and Wang et al. (10, 11), the MAP at the end of bronchoscopy was found to be decreased in the HFNC group compared to the COT group. Furthermore, in a patient who underwent bronchoscopic alveolar lavage, the MAP decreased from the time of bronchoscope entry into the tracheal bifurcation to the end of the examination following the application of HFNC (41). These findings align with the results of our study.

### 4.7 This systematic evaluation has several limitations

(i) The small sample size of the 12 included papers, the lack of studies using NIV, and the fact that some of the outcome indicators were only mentioned in individual papers may have affected the efficacy of the test, leading to possible differences in the results. (ii) Most of the included literature is in English, which raises the possibility of incomplete inclusion and introduces language bias. (iii) The lack of standardized oxygen flow rates and devices between the two groups in each study contributes to heterogeneity in some analyses, posing challenges for the implementation of clinical practice. (iv) The inclusion of studies from different countries with diverse characteristics may influence the efficacy of the analysis and hinder the broader applicability of the findings.

## 5 Conclusion

In summary, the application of HFNC in patients undergoing bronchoscopy demonstrated a significant improvement in reducing the incidence of hypoxemia and achieving higher minimum SpO_2_ levels compared to COT. However, HFNC showed less efficacy in comparison to NIV. Additionally, HFNC was found to be effective in reducing the duration of bronchoscopy and lowering PaO_2_ levels at the conclusion of the procedure. However, due to the limited number of studies and their lower quality included in the present meta-analysis, the overall effect remains uncertain. Therefore, further well-designed, multicenter, and high-quality RCTs are needed to establish the efficacy and safety of HFNC in patients undergoing bronchoscopy. It is imperative to conduct more scientifically rigorous RCTs with larger sample sizes and multicenter participation to further validate the efficacy and safety of HFNC in this context.

## Data availability statement

The raw data supporting the conclusions of this article will be made available by the authors, without undue reservation.

## Author contributions

CW: Data curation, Methodology, Software, Writing – original draft, Writing – review & editing. SM: Conceptualization, Data curation, Investigation, Writing – original draft. JW: Writing – original draft. NY: Writing – original draft. DW: Writing – original draft. LY: Funding acquisition, Project administration, Supervision, Writing – original draft. YW: Data curation, Formal analysis, Methodology, Project administration, Software, Supervision, Validation, Writing – original draft, Writing – review & editing.
